# *Si-CSP9* regulates the integument and moulting process of larvae in the red imported fire ant, *Solenopsis invicta*

**DOI:** 10.1038/srep09245

**Published:** 2015-03-18

**Authors:** Daifeng Cheng, Yongyue Lu, Ling Zeng, Guangwen Liang, Xiaofang He

**Affiliations:** 1 Department of Entomology, South China Agricultural University, Guangzhou, Guangdong, People's Republic China

## Abstract

Chemosensory proteins (CSPs) have been predicted to be involved in development; however, direct evidence for their involvement is lacking, and genetic basis is largely unknown. To determine the function of the chemosensory protein 9 (*Si-CSP9*) gene in *Solenopsis invicta,* we used RNA interference to silence *Si-CSP9* in 3rd-instar larvae. The 3rd-instar larvae failed to shed their cuticle after being fed *Si-CSP9*-directed siRNA, and expression profiling of RNAi-treated and untreated control larvae showed that 375 genes were differentially expressed. Pathway enrichment analysis revealed that 4 pathways associated with larval development were significantly enriched. Blast analysis revealed that one fatty acid amide hydrolase (*FAAH*) gene was up-regulated and 4 fatty acid synthase (*FAT*) genes and one protein kinase DC2 gene (*PKA*) were down-regulated in the enriched pathways. Significantly higher expression of these genes was found in 4th-instar larvae, and Pearson correlation analysis of the expression patterns revealed significant relationships among *Si-CSP9, PKA, FAAH*, and *FAT1-4*. Moreover, we confirmed that expression levels of *Si-CSP9, FAAH,* and *FAT1-4* were significantly reduced and that the development of 3rd-instar larvae was halted with *PKA* silencing. These results suggest that *Si-CSP9* and *PKA* may be involved in the network that contributes to development of 3rd-instar larvae.

Chemosensory proteins (CSPs) are a family of small, soluble proteins that are also referred to as OS-D-like^1^ or sensory appendage proteins^2^. Similarly to odorant-binding proteins (OBPs), CSPs are involved in solubilising and transporting pheromones through the aqueous haemolymph in insects. However, CSPs have an earlier origin than OBPs, as aqueous Arthropoda utilised a generic gene family of binding proteins (proto-CSPs) with diverse physiological roles prior to the colonisation of hostile terrestrial environments using OBPs^3^.

Research suggests that, similar to OBPs^4^, CSPs mainly function in olfaction and gustation by transporting hydrophobic ligands in the sensillum lymph in insects^5^,^6^. The CSP gene family exhibited lineage-specific expansions, with a large number of orthologous groups, over a short evolutionary time; however, these gradually disappeared with increasing divergence^7^. In addition, a higher copy number of CSPs is found in ants and other social insects than in non-social insects^7^. In recent years, however, many CSPs have been isolated from non-chemosensory organs, which indicates that CSPs have varied functions^2^,^8^,^9^,^10^,^11^.

As an invasive social insect^12^, the red imported fire ant (*Solenopsis invicta*) has been found to have a highly sophisticated chemosensory system^13^. A large number of genes and their biological functions have been determined following the sequencing of the genome of this species^14^, and thus far, a large number of CSPs, with 23 *Si-CSP* genes including 2 pseudogenes, have been found^7^. Although many studies have been performed on the chemosensory system of ants^9^,^15^,^16^,^17^,^18^,^19^, there are also a great number of genes for which the functions cannot be inferred from sequence alone^20^. Copy number variation has been suggested to have a significant role in adaptation and could be a starting point for the generation of genes with new functions^21^,^22^. It is surprising that, of the CSP genes, only *Si-CSP9* (accession number: EE129471) in *S. invicta* belongs to a distinct clade^9^, namely, that of Am-CSP5, which plays a role in the development of the embryonic integument in the honeybee^23^. Therefore, our hypothesis is that *Si-CSP9* functions during the integument and moulting process in *S. invicta* larvae.

In this study, we cloned the full-length *Si-CSP9* gene and identified its spatio-temporal expression patterns. To develop RNA interference (RNAi) for the 3rd-instar larvae of this ant, we used reverse genetics to validate directly whether a gene is essential during moulting. By comparing the larval structure and expression in the unsilenced and *Si-CSP9*-silenced samples, we illustrate the function of *Si-CSP9* and validate its relationship with the process of larval development.

## Results

### The structure of *Si-CSP9*

The 1,045 bp full-length *Si-CSP9* mRNA was obtained by RACE. The gene encoding *Si-CSP9* is relatively small, consisting of two exons, and most of the sequence from ATG to the STOP codon is occupied by one 784 bp intron.

### Expression patterns of *Si-CSP9* in developmental stages, castes and tissues

**Figure 1:**
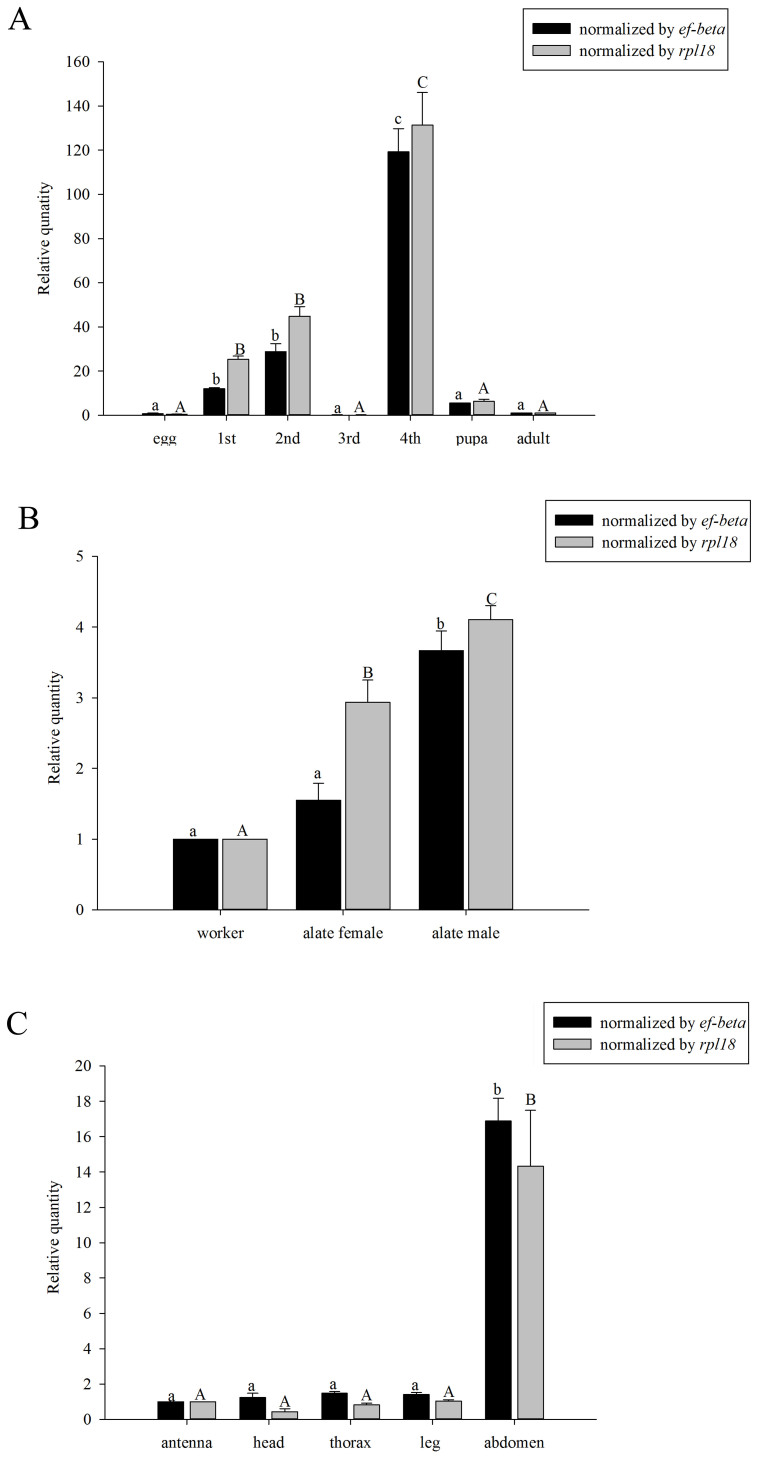
Expression of *Si-CSP9* in developmental stages, castes and tissues. (A): expression of *Si-CSP9* in eggs, 1st-instar larvae, 2nd-instar larvae, 3rd-instar larvae, 4th-instar larvae, pupae and adults; (B): expression of *Si-CSP9* in worker, alate females and alate males; (C): expression of *Si-CSP9* in the antenna, head, thorax, leg and abdomen of workers. Means ± SE that are labelled with the same letter within each treatment are not significantly different.

The expression level of *Si-CSP9* was significantly higher in 4th-instar larvae (L4) than at other stages (*ef-beta*: ANOVA, *F* = 144.686, *P* < 0.001; *rpl18*: *F* = 66.529, *P* < 0.001 Figure 1A). When investigated in different castes, significantly higher expression was observed in the alate females than in the other castes (*ef-beta*: ANOVA, *F* = 43.714, *P* < 0.001; *rpl18*: *F* = 53.505, *P* < 0.001, Figure 1B). Furthermore, the expression of *Si-CSP9* in the tissues of workers was investigated. Surprisingly, *Si-CSP9* exhibited significantly higher expression in the abdomen than in the olfactory tissues (antennae) (*ef-beta*: ANOVA, *F* = 14.478, *P* < 0.001; *rpl18*: *F* = 18.192, *P* < 0.001, Figure 1C), whereas other *Si-CSP* genes are specifically expressed in the olfactory organs^9^.

### Expression profiles of *Si-CSP9* during developmental process between L3 and L4

**Figure 2:**
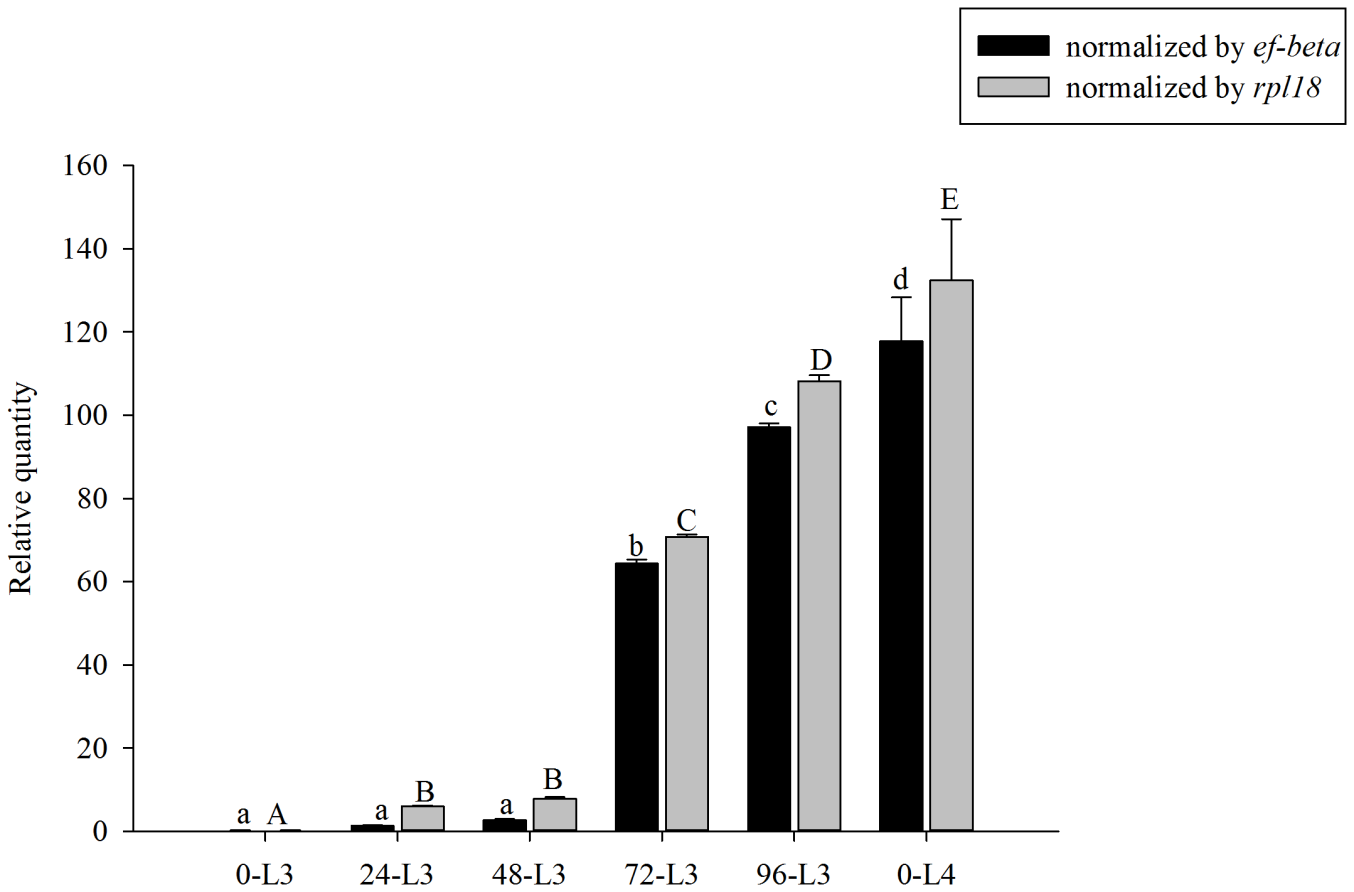
Expression of *Si-CSP9* during developmental process between L3 and L4. 0-L3: newly emerged 3rd-instar larvae; 24-L3: 24-h-old 3rd-instar larvae; 48-L3: 48-h-old 3rd-instar larvae; 72-L3: 72-h-old 3rd-instar larvae; 96-L3: 96-h-old 3rd-instar larvae; 0-L4: 4th-instar larvae. Means ± SE that are labelled with the same letter within each treatment are not significantly different.

By investigating the expression profiles of *Si-CSP9* during the developmental process between 3rd-instar larvae (L3) and 4th-instar larvae (L4), *Si-CSP9* was found to be significantly more highly expressed in 72-h and 96-h L3 (when L3 begin to moult) (*ef-beta*: ANOVA, *F* = 5521.30, *P* < 0.001; *rpl18*: *F* = 3991.028, *P* < 0.001, Figure 2).

### Functional investigation of *Si-CSP9* by RNAi

**Figure 3:**
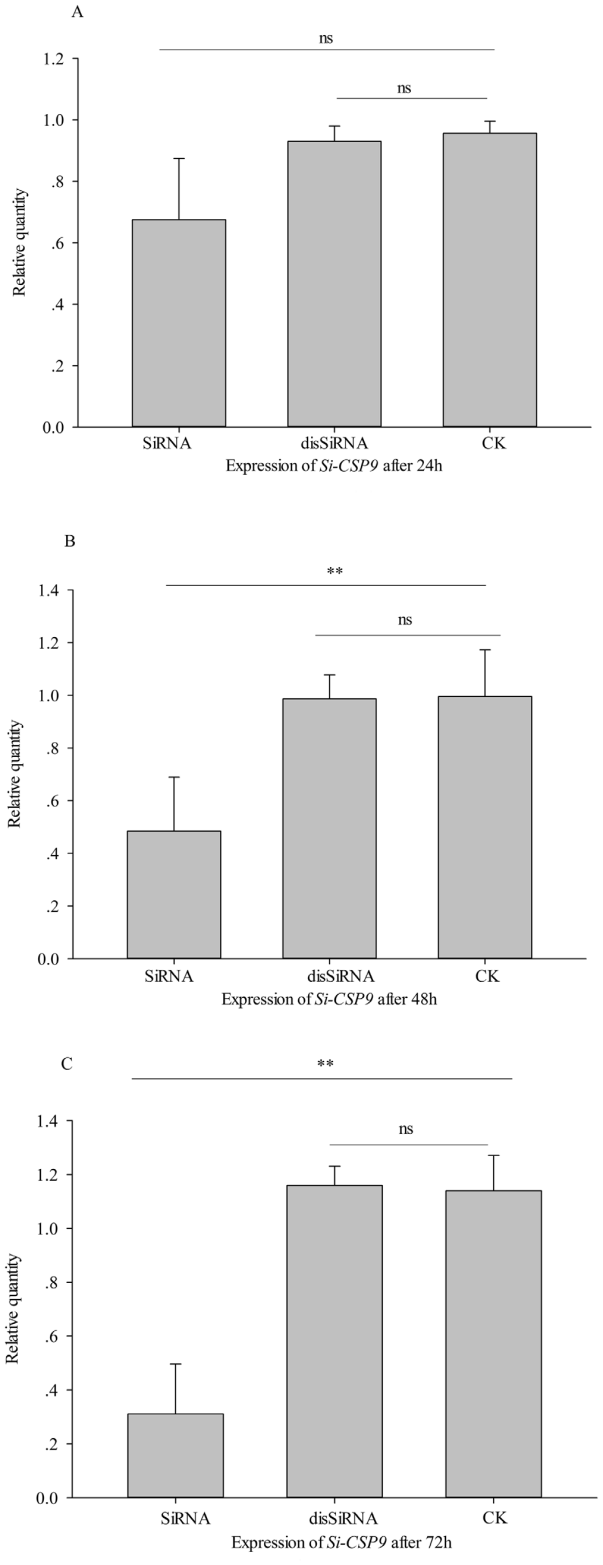
Expression of *Si-CSP9* after silencing. (A): Expression of *Si-CSP9* after being silenced for 24 h. (B): Expression of *Si-CSP9* after being silenced for 48 h. (C): Expression of *Si-CSP9* after being silenced for 72 h. SiRNA: small interference RNA; disSiRNA: small interference RNA with a disordered sequence; CK: sugar-water. In all groups, for treatment compared with CK, “**” denotes *P* < 0.01; “ns” denotes not significant.

We designed an RNAi assay targeting the *Si-CSP9* gene in L3 and investigated the temporal dynamic gene expression of *Si-CSP9* and phenotype changes after RNAi treatment. Twenty-four hours after RNAi treatment, the expression of *Si-CSP9* exhibited no significant difference in the SiRNA and disSiRNA samples and in a 10% sugar-water feeding treatment (normal control treatment, CK) (independent samples t-test, *t = * 2.392, 0.738, *P = * 0.075, 0.502, respectively, Figure 3A). After 48 h, the expression of *Si-CSP9* exhibited a significantly lower level in SiRNA than in CK (independent samples t test, *t = * 3.263, *P = * 0.031, Figure 3B), whereas no significant difference was found between disSiRNA and CK (independent samples *t* test, *t* = 0.075, *P* = 0.943, Figure 3B). At 72 h, the expression of *Si-CSP9* had decreased significantly to approximately 23.7% of CK (independent samples t test: *t = * 6.325, *P =* 0.003, Figure 3C), although no significant difference was observed between disSiRNA samples and CK samples (independent samples *t* test *t = * −0.272, *P = * 0.799, Figure 3C). Moreover, no differences were found for *Si-CSP2* and *Si-CSP3* expression among the SiRNA, disSiRNA and CK samples (*Si-CSP2*: independent samples t test, SiRNA vs. CK, 24 h, *t* = 0.372, *P* = 0.728; 48 h, *t* = 0.742, *P* = 0.499; 72 h, *t* = 0.612, *P = * 0.574. disSiRNA vs. CK, 24 h, *t* = 0.414, *P* = 0.7; 48 h, *t* = 0.477, *P* = 0.658; 72 h, *t* = 1.018, *P = * 0.366) (*Si-CSP3*: independent samples t test, SiRNA vs. CK, 24 h, *t* = 0.74, *P* = 0.499; 48 h, *t* = 0.742, *P* = 0.499; 72 h, *t* = −0.372, *P = * 0.728. disSiRNA vs. CK, 24 h, *t* = 0.477, *P* = 0.658; 48 h, *t* = 1.21, *P* = 0.29; 72 h, *t* = 0.414, *P = * 0.7) (Supplementary Figure 1).

**Figure 4:**
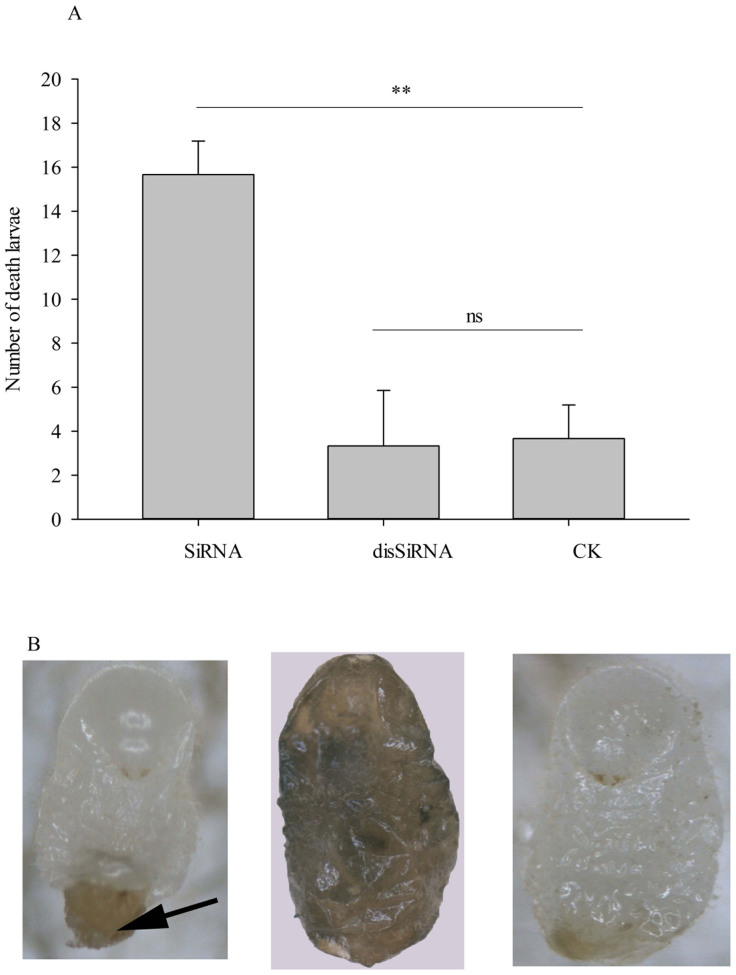
Mortality and phenotype of larvae after silencing of *Si-CSP9*. (A): Number of dead larvae after silencing of *Si-CSP9*. (B): Phenotype of larvae after silencing of *Si-CSP9*. In all groups, for treatment compared with CK, “**”denotes *P* < 0.01; “ns” denotes not significant.

An investigation of larval mortality showed that L3 fed the *Si-CSP9* SiRNA had a significantly higher mortality than CK (independent samples *t =* 9.62, *P = * 0.001, Figure 4A); no significant difference between the disSiRNA feeding treatment and CK was found (independent samples *t = * 0.2, *P = * 0.85, Figure 4A). Moreover, it appeared that although the 3rd-instar larvae were viable and began their development towards L4, they failed to shed the 3rd-instar larval cuticle in the last phase of the moulting process, ecdysis, which resulted in the old cuticle remaining attached to the partially moulted body (as shown in Figure 4B). Most of the dead larvae were found to exhibit a brown nodule (as shown by the black arrow in Figure 4B, left) on the abdomen. Body shrinking and melanism were also found for the dead larvae (Figure 4B middle) in the SiRNA feeding treatment, whereas the larvae developed normally into L4 in CK (Figure 4B right).

### Transcription expression profile after *Si-CSP9* down-regulation

**Figure 5:**
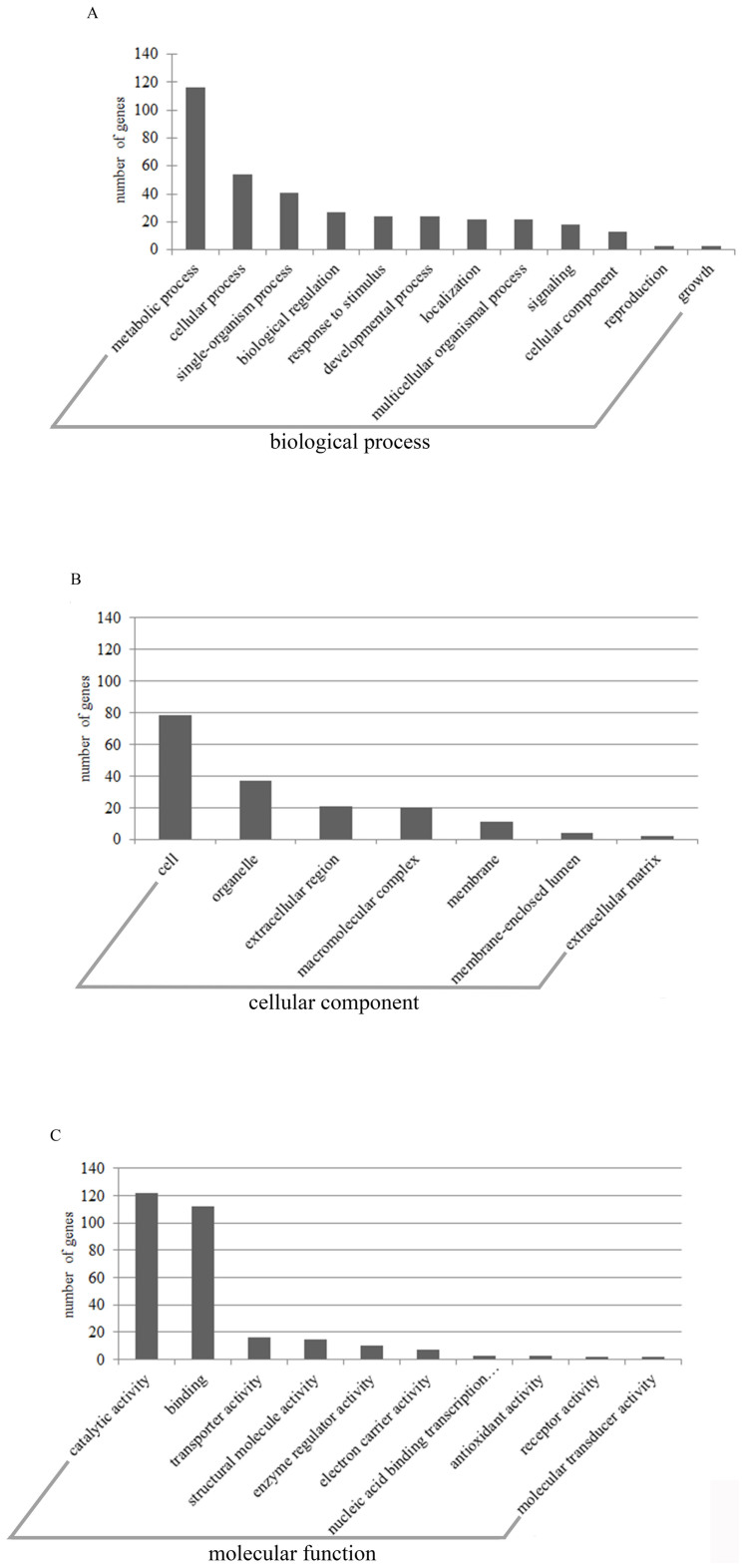
Diversity of ontological categories of differentially expressed genes. (A): Ontological categories in biological process. (B): Ontological categories in cell component. (C): Ontological categories in molecular function.

**Figure 6:**
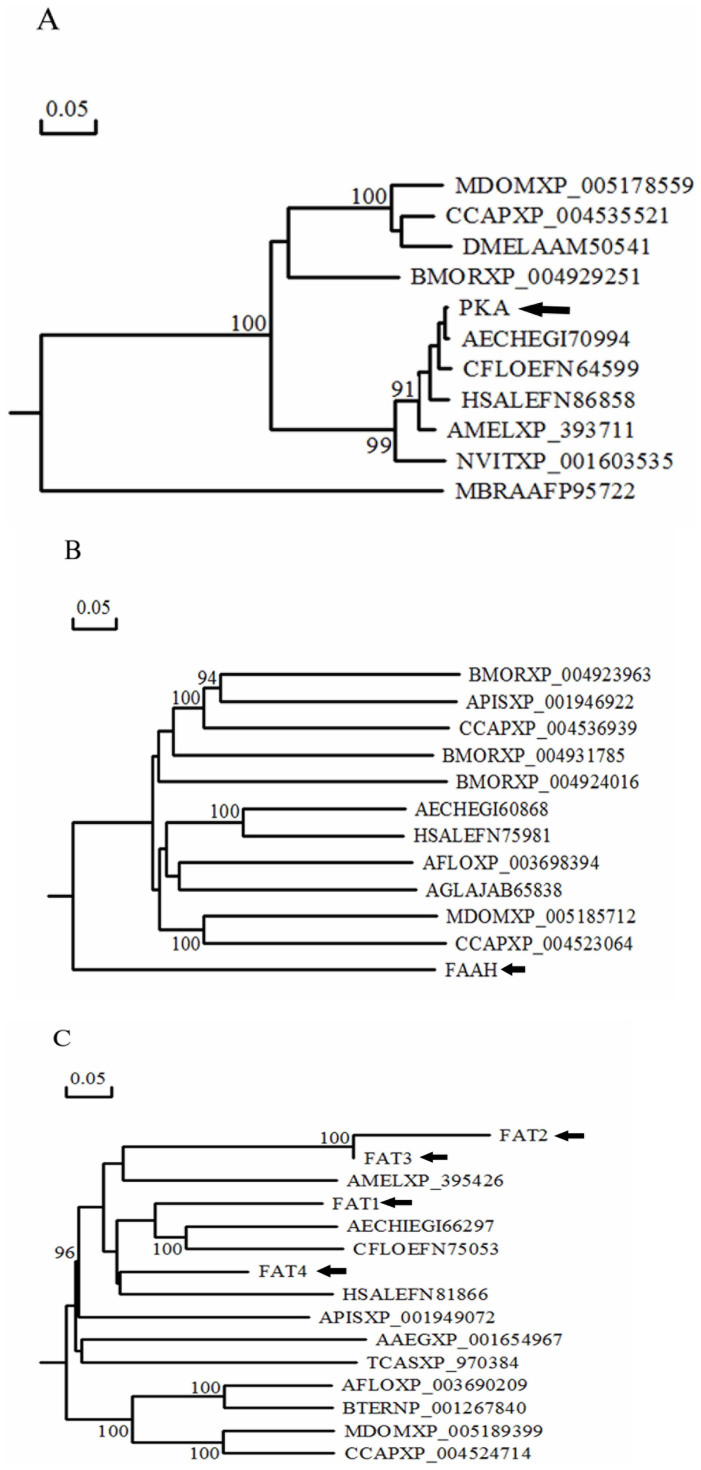
Neighbour-joining trees of *PKA, FAAH,* and *FAT1-4* created using DNAMAN software. The tree is collapsed to nodes with 50% or greater bootstrap support (n = 1000 replicates), and the bootstrap values are listed at each node. (A): Neighbour-joining tree of *PKA.* (B): Neighbour-joining tree of *FAAH*. C: Neighbour-joining tree of *FAT1-4*. Information on the genes is given in Supplementary Table S1.

There were 375 differentially expressed genes (67 up-regulated genes and 308 down-regulated genes) when *Si-CSP9* was silenced in the SiRNA feeding treatment. These genes fell into various ontological categories (Figure 5) and pathways (Table 1). With regard to biological processes for the differentially expressed genes, the metabolic process exhibited the highest number of differentially expressed genes, at 116 (Figure 5A, Supplementary Dataset S1). The genes in the metabolic process were significantly enriched in the following: fatty acid biosynthesis; glycine, serine and threonine metabolism; metabolic pathways; the hedgehog signalling pathway; and carbon-nitrogen ligase activity, with glutamine as amido-N-donor (Table 1). As has been previously reported, fatty acid biosynthesis, metabolic pathways, hedgehog signalling, and glutamine amido-N-donor carbon-nitrogen ligase activity have been demonstrated to have a significant effect on the development of insect larvae^24^,^25^,^26^,^27^,^28^. We performed blast and phylogenetic analyses of the most differentially expressed genes in the significantly enriched pathways and metabolic processes: namely, a protein kinase DC2 gene (*PKA*) involved in the hedgehog signalling pathway, one fatty acid amide hydrolase gene (*FAAH*) involved in glutamine amido-N-donor carbon-nitrogen ligase activity, and 4 fatty acid synthase genes (*FAT1, FAT2, FAT3, FAT4*) involved in the fatty acid biosynthesis and metabolic pathways (Figure 6A, 6B, and 6C; Table 1, Table 2).

### Relationship between expression patterns of *Si-CSP9*, *PKA*, *FAAH*, and *FAT1-4* and developmental stages

**Figure 7:**
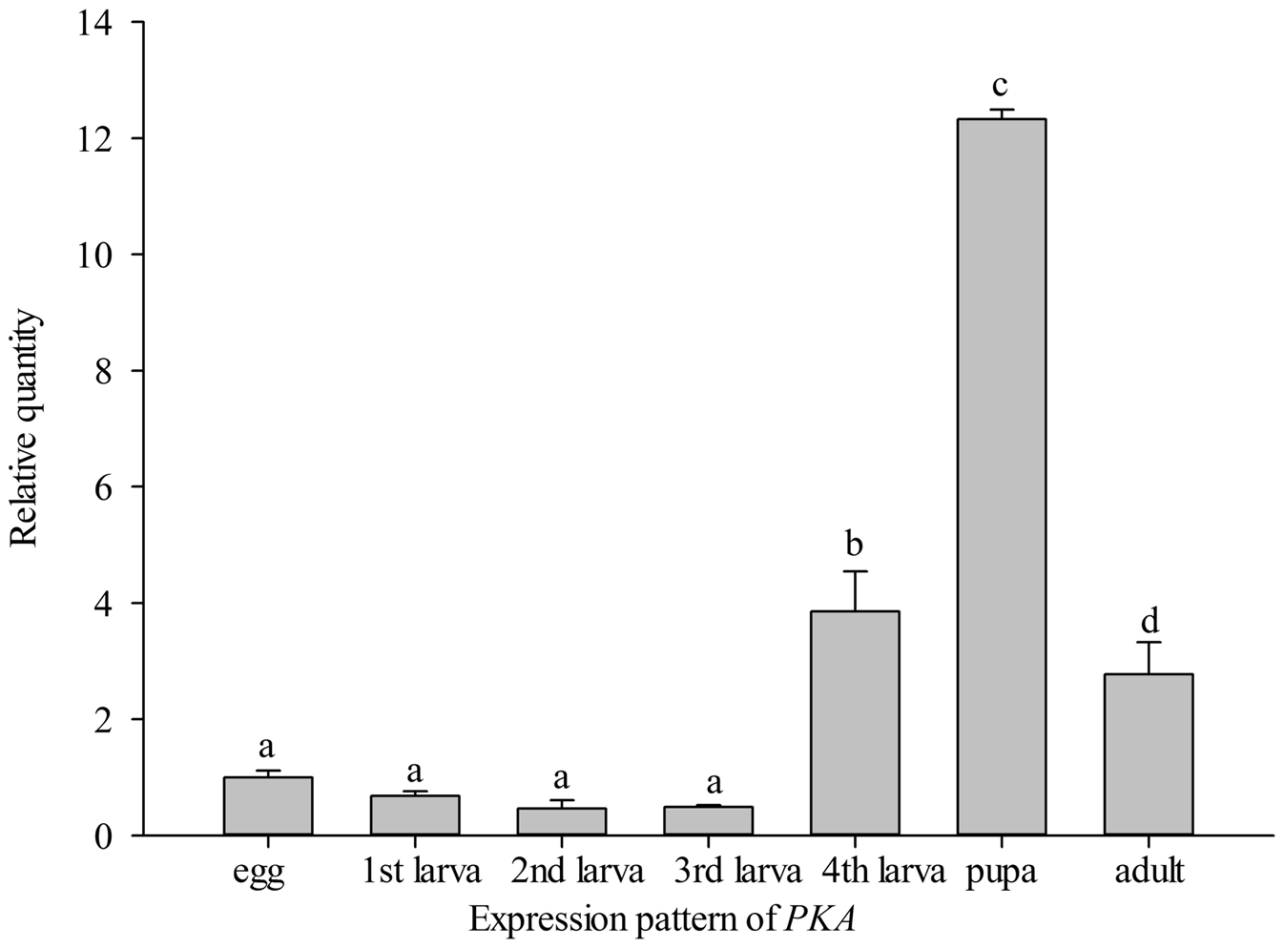
Development-specific expression of *PKA*. Means ± SE that are labelled with the same letter within each treatment are not significantly different.

**Figure 8:**
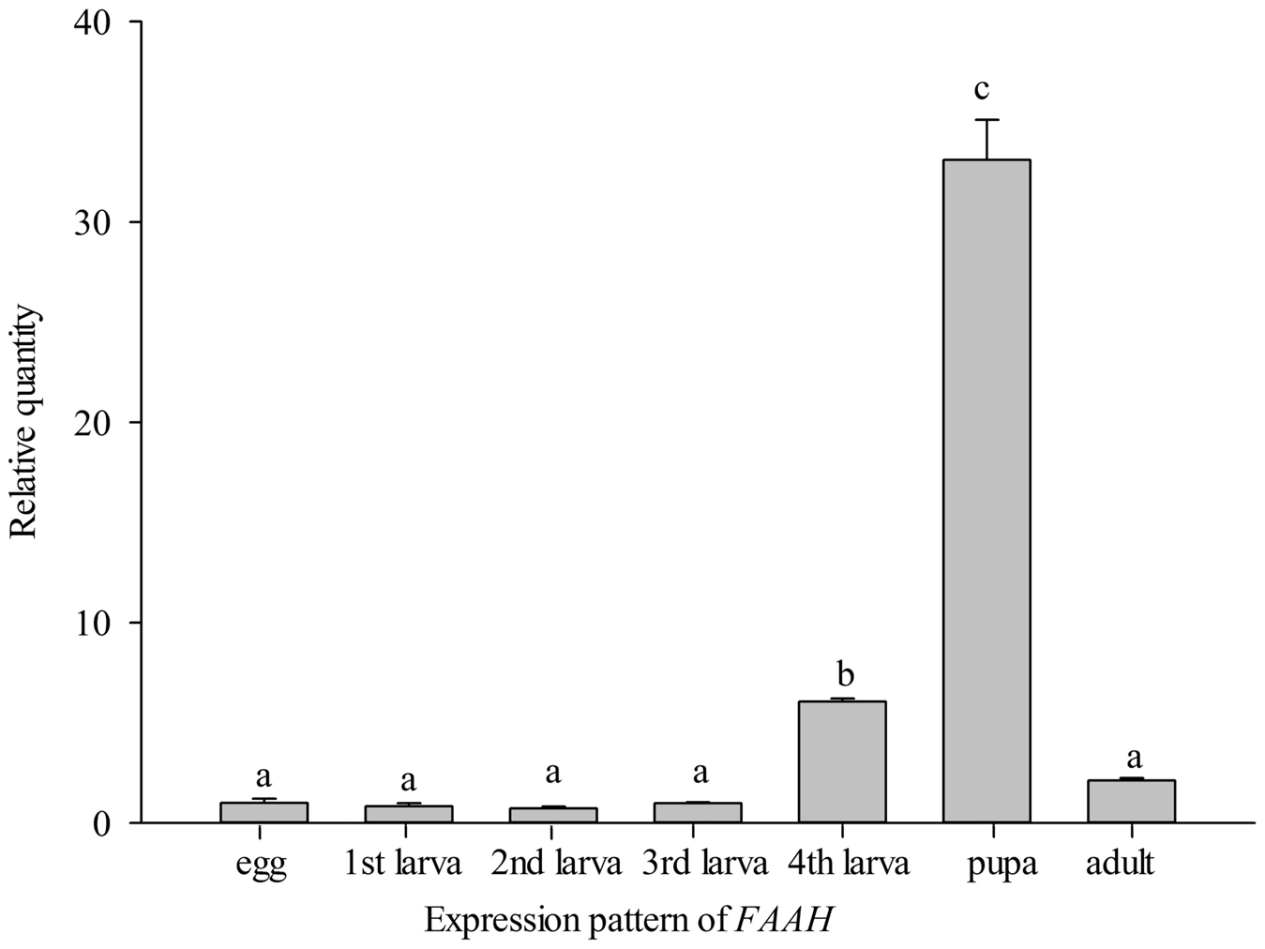
Development-specific expression of *FAAH*. Means ± SE that are labelled with the same letter within each treatment are not significantly different.

**Figure 9:**
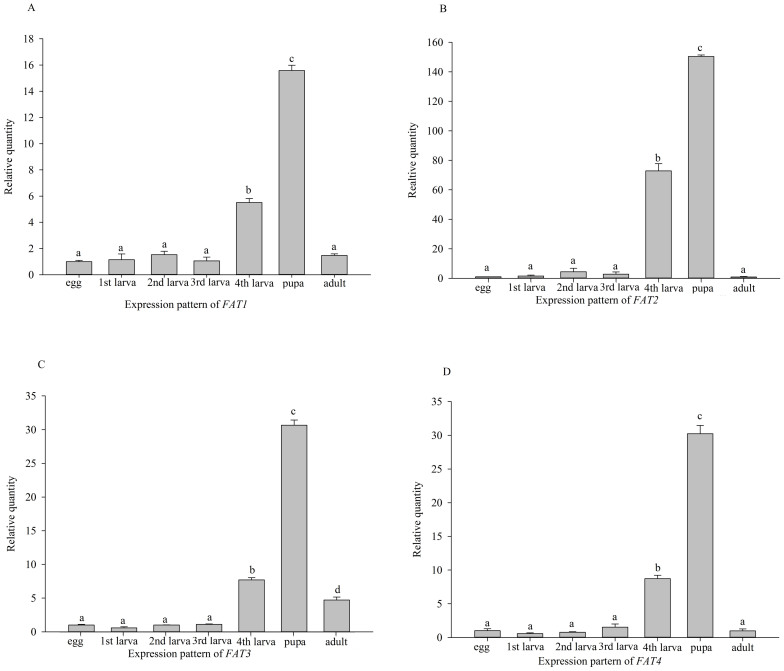
Development-specific expressions of *FAT1-4*. Means ± SE that are labelled with the same letter within each treatment are not significantly different.

A one-way analysis of variance showed that *PKA*, *FAAH*, and *FAT1-4* had significantly higher expression in 4th-instar larvae than other developmental stages, identical to *Si-CSP9* (*PKA*: *F =* 461.68, *P <* 0.001, Figure 7; *FAAH*: *F* = 719.24, *P* < 0.001, Figure 8; *FAT1*: *F = *1001, *P <* 0.001; *FAT2*: *F =* 2127, *P <* 0.001; *FAT3*: *F* = 2701, *P <* 0.001; *FAT4*: *F* = 1222, *P <* 0.001, Figure 9). The expression pattern correlation analysis between *PKA*, *FAAH*, *FAT1-4*, and *Si-CSP9* revealed the same expression patterns in the developmental stages, with each gene exhibiting a significantly related Pearson correlation with another gene at the 0.01 level (Table 3).

### Functional analysis of *PKA* by RNAi

**Figure 10:**
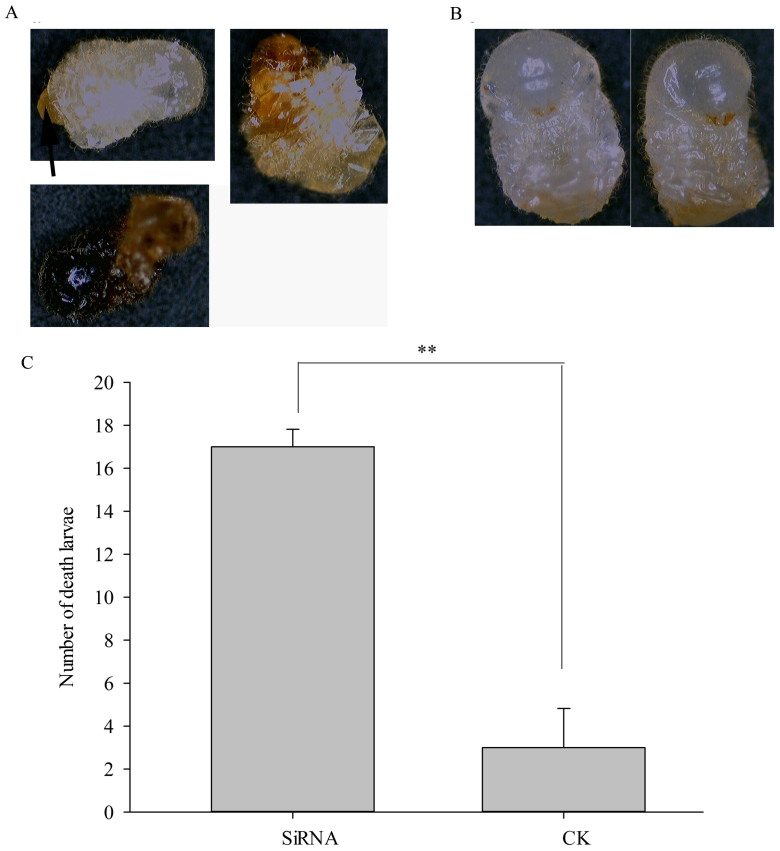
Phenotype and mortality of larvae after *PKA* silencing. (A): Phenotype of larvae with *PKA* silencing. (B): Phenotype of larvae in CK. (C): Number of dead compared within the silenced *PKA* samples and normally expressed *PKA* samples. “**” denotes *P* < 0.01.

Phenotypic observations revealed that the larvae treated with *PKA* RNAi had a slower development rate compared to the normal control treatment. The larvae were unable to moult or moulted incompletely under *PKA* RNAi treatment, and this dramatic change was found after 72 h of RNAi feeding (Figure 10). Separation between the 3rd-instar larval cuticle and the newly synthesised 4th-instar larval cuticle was observed. However, the L4 could not remove the cuticle from their bodies, and it remained attached to the abdomen by a brown nodule (Figure 10A, black arrow). The other type of deformation was characterised by an atrophic body (Figure 10A, right); the ultimate destiny of the larvae was death and melanism (Figure 10A, bottom). In contrast, the larvae receiving the normal control treatment displayed all the normal characteristics of L4 and developed normally and successfully to the next stage (Figure 10B). All these phenotypes were identical to those in the *Si-CSP9* RNAi treatment (Figure 4).

The difference in mortality of L3 between the RNAi treatment and normal control treatment was analysed after 96 h, with a significantly higher mortality observed under the RNAi treatment (independent samples t-test, *t =* −14, *P <* 0.001; Figure 10C). This result was similar to the result obtained under the *Si-CSP9* RNAi treatment (Figure 4).

### Effects of *PKA* RNAi treatment on the expression of *Si-CSP9*, *FAAH*, and *FAT*s

**Figure 11:**
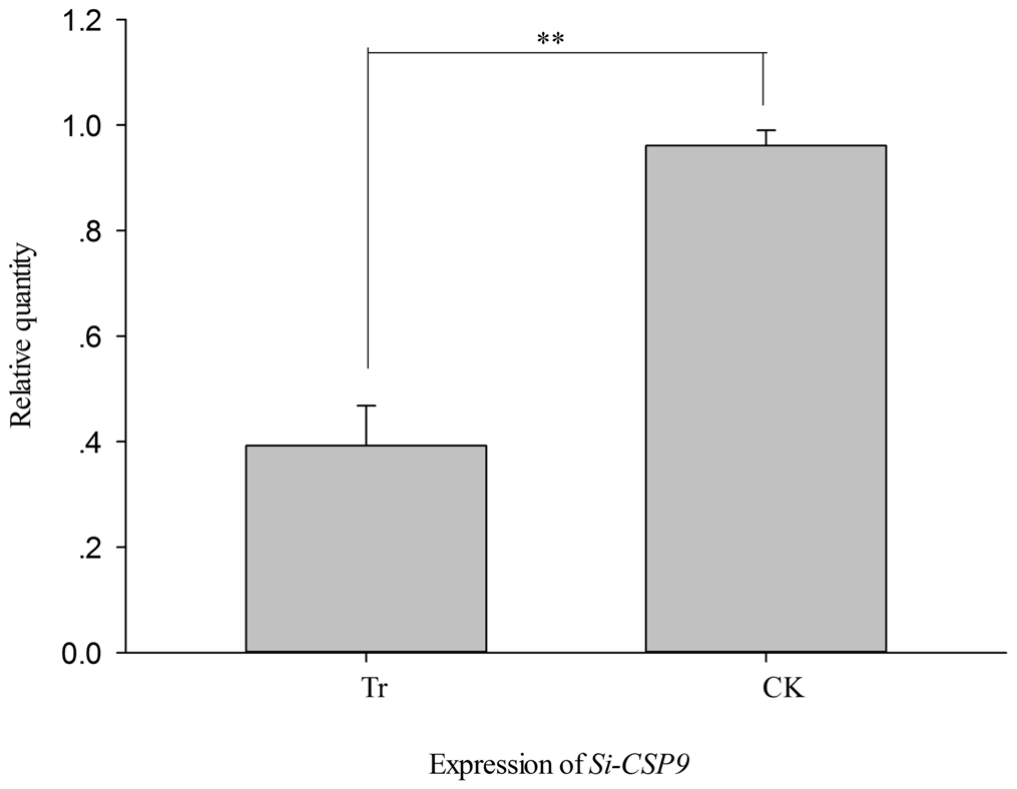
Effects on expression of *Si-CSP9* after silencing of *PKA*. Tr: samples with PKA silenced; CK: samples with *PKA* normally expressed.

**Figure 12:**
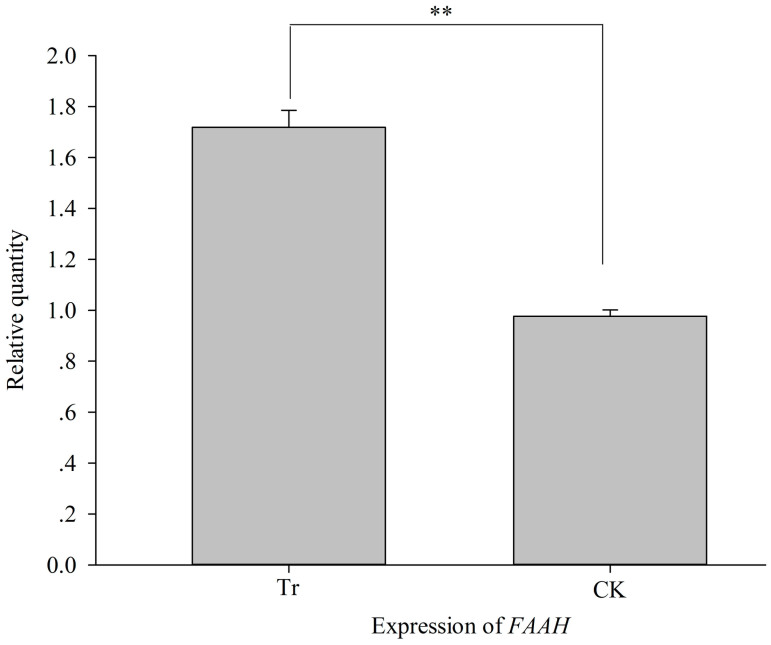
Effects on expression of *FAAH* after silencing of *PKA*. Tr: samples with PKA silenced; CK: samples with *PKA* normally expressed.

**Figure 13:**
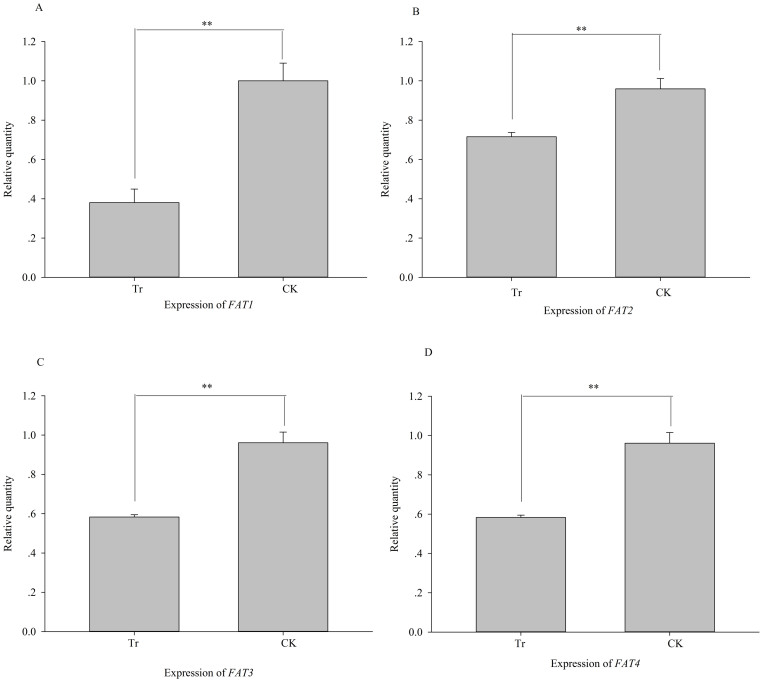
Effects on expression of *FAT1-4* after silencing of *PKA*. (A): expression of *FAT1*; (B): expression of *FAT2*; (C): expression of *FAT3*; (D): expression of *FAT4*. Tr: samples with PKA silenced; CK: samples with *PKA* normally expressed.

In the *PKA* RNAi treatment, *Si-CSP9* and *FAT1-4* exhibited a significant decrease in expression, whereas *FAAH* increased significantly, consistent with the *Si-CSP9* RNAi treatment (independent samples t-test, *Si-CSP9*: *t = * −12.137, *P <* 0.001, Figure 11; *FAAH*: *t = * −18.12, *P <* 0.001, Figure 12; *FAT1*: *t =* −15.566, *P <* 0.001; *FAT2*: *t = * −7.435, *P* = 0.002; *FAT3*: *t = * −16.142, *P <* 0.001; *FAT4*: *t = * −11.78, *P <* 0.001, Figure 13).

## Discussion

In this study, we demonstrated that the chemosensory protein encoded by *Si-CSP9* may be involved in the developmental process that occurs in the transition from L3 to L4, particularly the cuticularisation and moulting processes, in which *FAAH* and *FAT1-4* play important roles^29^,^30^.

Through RNAi assays and oligonucleotide microarray analysis, we illustrated that decreased *Si-CSP9* expression may affect fatty acid biosynthesis, metabolic pathways, and glutamine amido-N-donor carbon-nitrogen ligase activity. Indeed, *FAT*s and *FAAH* are differentially expressed, affecting the development of larvae (Table 1 & Table 2), though the disSiRNA control showed no such effects (data not shown). To our knowledge, this study is the first to identify the relationship between chemosensory genes and *FAAH* and *FATs* genes in the red imported fire ant, and our data are important for understanding the new role of chemosensory proteins in social insects.

Recently, animal development has been found to be affected by many internal and external factors^31^,^32^,^33^. Previous studies have also indicated similar roles of the chemosensory system in other organisms. For example, studies have indicated that chemosensory neurons^34^ and small-molecule pheromones in the nematode *Caenorhabditis elegans* can control larval development. Data also show that *C. elegans* larval development is controlled by the activities of four classes of chemosensory neurons and that larvae are regulated by competing environmental stimuli: food and a dauer pheromone, which are recognised by chemosensory proteins^35^,^36^. Interestingly, studies have also found a novel role for chemosensory proteins in controlling development in insects^23^. CSPs from other insects have been shown to be expressed in large amounts in the epidermis^11^,^37^,^38^, which is critical during development^39^,^40^. However, more evidence is required to determine whether there are environmental stimuli that can be recognised by *Si-CSP9* and affect the development of red imported fire ant larvae.

Similar techniques and results have been reported in the honeybee (*Apis mellifera*)^23^. Using RNAi, researchers have found that CSP5 plays a role in the development of the embryonic integument. However, no analysis of the RNAi-induced phenotype by high-throughput technologies such as microarrays to identify other genes involved in this transition has been performed. In our experiments, the function of *Si-CSP9* in the developmental process between L3 and L4 was revealed by RNAi. Moreover, the entire network of interactions during cuticle synthesis was unravelled using RNA-seq. Our results clearly provide evidence for the hypothesis that CSPs may perform a non-olfactory function, which will be essential to understanding the origins of evolutionary novelties in different lineages. However, the affected stages are different between the honeybee and red imported fire ant. This result demonstrates the multifunctional nature of CSPs, especially in Hymenoptera.

Furthermore, a gene identified in our study, *PKA*, is involved in the hedgehog signalling pathway, which plays key roles in a wide variety of developmental processes, even in larval body segment development and in the formation of adult appendages^41^. Abnormal larvae and a significantly higher death rate, which were also observed with *Si-CSP9* RNAi treatment, were observed with *PKA* silencing (Figure 4 & Figure 10). Thus, we predict interaction between *Si-CSP9* and *PKA* in the larval development of *S. invicta*. Fujiwara, et al. (2002)^42^ have found that interaction between sensory stimuli and *PKA* can regulate the body size and behavioural state of *C. elegans*, which is direct evidence that *PKA* has a close and vital relationship with the chemosensory system during olfactory recognition. We also found that *Si-CSP9* was down-regulated with *PKA* silencing (Figure 11). Furthermore, the correlation analysis of expression patterns between *Si-CSP9* and *PKA* in developmental stages showed a significant relationship (Table 3). Thus, we suggest that *Si-CSP9* and *PKA* are involved in the same network that affects larval development.

Our results also suggested that *FAAH* and *FATs* are significantly affected by *PKA* silencing, with an abnormal phenotype (Figure 10, Figure 12 and Figure 13). *PKA* exhibited a significant Pearson correlation with *FAAH* and *FATs* with regard to expression patterns (Table 3). As the primary element^43^ in the cAMP signal transduction system, one of several second messenger-dependent pathways that generates intracellular responses to extracellular signals^44^, *PKA* was also found to affect the development of *Drosophila* larvae^45^. Studies have found that interactions between the inositol and cyclic AMP signalling pathways, in which the role of *PKA* is important, can regulate larval moulting in *Drosophila*^46^. In addition, *PKA* activity is regulated by chemosensory stimulation in the honeybee antennal lobe^47^. All this evidence leads us to believe that *PKA* can regulate larval development in the red imported fire ant.

However, the details of the interaction between *Si-CSP9* and *PKA* remain unclear. Research indicates that the cAMP/*PKA* pathway rapidly activates SIRT1 to promote fatty acid oxidation independently of changes in NAD^+^ 48. As mentioned by Maleszka (2007)^23^, it is reasonable to assume that *Si-CSP9* encodes a carrier protein transporting lipophilic compounds used for embryonic integument synthesis, a role consistent with the properties of CSPs. Hydrocarbons (HCs), which are synthesised by oenocytes situated in the integument, comprise one of the major constituents of the insect epicuticular lipid layer^49^. The cuticle and ovary appear to be the main target tissues for the transport pathways of insect HCs^49^. Research has also found that interactions between cuticular hydrocarbon and CSPs are vital in ant nestmate and non-nestmate discrimination^50^. Thus, it is possible that *Si-CSP9* and *PKA* are involved in the shuttling of HCs through an aqueous medium to the epicuticle. However, more data are needed to examine this hypothesis.

Traditionally, CSPs are thought to function in olfaction and gustation by transporting hydrophobic ligands in the sensillum lymph^5^,^6^. Our studies suggested that the *CSP*s could play a different role, controlling the development of larvae by affecting the expression of *PKA*, *FAAH*, and *FAT*s. Indeed, we identified several gene categories that are candidates for controlling the development of larvae and have close relationships to *Si-CSP9*, and this molecular mechanism is particularly significant for understanding the novel function of the CSP family.

## Methods

### Insects

Three colonies (polygyne) of red imported fire ants were collected from the campus of South China Agriculture University, Guangzhou, China (23.150967N, 113.3552E) and placed in plastic boxes with the walls dusted with talcum powder. The ant colonies were maintained in an incubator with 80% humidity, 26 ± 2°C and a 12:12 dark/light photoperiod and reared with 10% sugar-water and *Tenebrio molitor*.

### Experimental samples

For each colony, samples of insects at newly emerged developmental stages (egg, 1st-instar larvae, 2nd-instar larvae, 3rd-instar larvae (L3), 4th-instar larvae (L4), pupae and adults), castes (females, males and workers) and tissues (antennae, heads, thoraxes, legs and abdomens) of workers were collected and immediately place in liquid nitrogen for later qRT-PCR. For developmental stages and castes, 5 ants were selected for each sample; for tissues, 100 ants were dissected for each sample. Three replicate samples were taken for each stage, caste and tissue. Nine individuals (3 individuals for each sample) were also sampled every 24 hours during the L3 and L4 stages to investigate the expression profiles of *Si-CSP9*.

### RNA extraction and quality assessment

Total RNA was extracted using the TRIzol reagent (Invitrogen, USA) following the manufacturer's instructions. The RNA sample quality was examined through 4 steps: (1) analysis of sample degradation and contamination via agarose gel electrophoresis; (2) examination of purity using a NanoDrop 2000 spectrophotometer; (3) precise quantification of the concentration using a Qubit® 2.0 fluorometer; and (4) accurate detection of integrity using an Agilent 2100 Bioanalyzer.

### 5′ race and 3′ race analysis of *Si-CSP9*

To determine the structure of *Si-CSP9*, 5′ and 3′ rapid amplification of cDNA ends (RACE) was performed using the SMARTer^TM^ RACE cDNA Amplification Kit (Clontech, California, USA) according to the manufacturer's instructions (primer sequences are shown in Table 4). To determine the exon and intron structure of this gene, the full-length cDNA of *Si-CSP9* was subjected to a nucleotide Blast search using *S. invicta* genomic resources (http://hymenopteragenome.org/ant_genomes/?q=blast).

### RNA interference (RNAi)

Small interference RNAs (SiRNA) specific to *Si-CSP9* and *PKA* were prepared using an in vitro transcription T7 kit (Takara) following the manufacturer's instructions (primer sequences are shown in Table 1). As a control, small interference RNAs with disordered sequences to the target genes (disSiRNA) were also prepared. RNAi and phenotype analyses were performed to identify the *in vivo* function of *Si-CSP9*. In this procedure, 12 μg siRNA complementary to *Si-CSP9* (siRNA) was mixed into sugar-water and fed to L3. As a control, 10% sugar-water and sequence-disordered siRNA (disSiRNA) mixed in sugar-water were also fed to L3. For 72 hours, the larvae were sampled every 24 hours to identify the expression of *Si-CSP9* by qRT-PCR. A control for the expression of CSPs during the RNAi experiment was confirmed by investigating the expression of *Si-CSP2* and *Si-CSP3*, which have the highest degree of homology to *Si-CSP9*^9^.

### RNA-seq and analysis of differentially expressed genes

To detect associations between *Si-CSP9* and other genes or pathways, gene expression profile differences between the *Si-CSP9* RNAi treatment sample and *Si-CSP9* normally control sample were compared by RNA-seq. The quantified RNA samples were enriched for mRNA using magnetic beads with oligonucleotide (dT), and the enriched mRNA was then fragmented into 400–600 bp fragments using fragmentation buffer and used as a template to synthesise both the first-strand cDNA and second-strand cDNA. The double-stranded cDNA generated was purified using AMPure XP beads, and the end of the double-stranded cDNA was then repaired, a base A tail was added, and sequencing adapters were connected to the end of the double-stranded cDNA. Finally, fragments were selected based on size (400–600 bp fragments) using AMPure XP beads. PCR was used for amplification, and the PCR products were purified using AMPure XP beads to generate cDNA libraries. The prepared libraries were sequenced by the pair-end method using the Illumina HiSeq platform. The sequencing read length was 200 bp. We sequenced 2.66 G clean bases for each sample. The Trinity software^51^ was used for transcript assembly (version: v2012-10-05; min_kmer_cov = 2; the default settings were used for the remaining parameters). The assembly process was as described in Grabherr (2011)^52^. The sequences assembled by Trinity were mapped onto the genome of the red imported fire ant (http://hymenopteragenome.org/ant_genomes/?q=blast) for the ensuing analysis. In the mapping process, the software RSEM was used according to the manufacturer's instruction^53^. The mapping results from RSEM were calculated to generate the read count for each gene and transferred into RPKM (reads per kilobases per million mapped reads) using the estimation method in Mortazavi et al. (2008)^54^. DESeq^54^ was introduced to analyse the read count data and identify differentially expressed genes under different experimental conditions. In the case of genes with FDR ≤ 0.001 and |log2Ratio| ≥ 1, the tested gene was differently expressed compared to the reference sample^54^,^55^.

Pathway enrichment analysis was performed using the KEGG Orthology-based Annotation System 2.0 (KOBAS 2.0, http://kobas.cbi.pku.edu.cn) in the *Drosophila melanogaster* database. InterPro categories were enriched for the supplied gene list based on the algorithm presented by GOstat^56^.

### Phenotype observation after *Si-CSP9* and protein kinase DC2 gene (*PKA*) silencing

Twenty L3 were selected and cared for by 15 adult workers in an incubator. Every 24 h, the ants were fed with 12 μg siRNA mixed in sugar-water (SiRNA) as the test treatment. Two groups of ants were used as controls: the first group received the control treatment, being fed only 10% sugar-water, and the second group was fed with 12 μg disSiRNA mixed in 10% sugar-water. After 72 h, we recorded the number of dead larvae and photographed them.

### Expression patterns and relationships between *Si-CSP9*, *PKA*, the fatty acid amide hydrolase (*FAAH*) gene, and fatty acid synthase (*FAT*) genes

To confirm the RNA-seq results, the expression levels of the four groups of significantly differentially expressed genes (*Si-CSP9*, *PKA*, *FAA* and *FAT1-4*) were detected by qRT-PCR. Newly emerged eggs, 1st-instar larvae, 2nd-instar larvae, 3rd-instar larvae, 4th-instar larvae, pupae and adults were collected, and total RNA was extracted. The cDNA was reverse-transcribed from 2 μg total RNA using PrimeScript^R^° 1^st^ Strand cDNA Synthesis Kit (Takara). The expression levels of *Si-CSP9*, *PKA*, *FAAH*, and *FAT1-4* were investigated by qRT-PCR (primer sequences are shown in Table 4)*.* The standard curve method was used to measure the relative expression levels of the samples, and *ef-beta* and *rpl18* were used as reference genes to normalise the reaction^57^. PCR amplification was conducted using the Mx3000P spectrofluorometric thermal cycler (Stratagene), as follows: a 2 min incubation at 95°C, followed by 40 cycles of 95°C for 20 s, 57°C for 30 s, and 68°C for 20 s. A melting curve analysis was performed to confirm the specificity of amplification.

### Statistical analysis

The independent samples t-test was applied to test the expression differences of *Si-CSP9* and *PKA* between SiRNA-fed ants and CK and the differences between the numbers of dead larvae in the SiRNA-fed ants and CK. Differences in the expression patterns of *Si-CSP9*, *PKA*, *FAAH*, and *FAT1-4* were compared by a one-way analysis of variance (ANOVA), followed by Tukey's test for multiple comparisons. Pearson correlation coefficients between the gene expression patterns were calculated and compared using the independent samples t-test. Differences were considered to be significant at *P* < 0.05. The data were analysed using SPSS 16.0.

## Author Contributions

D.C.: study design, experimental studies, statistical analysis and manuscript preparation. L.Z., Y.L. and G.L.: manuscript editing. X.H.: approval of the final version of the manuscript.

## Supplementary Material

Supplementary Information Supplementary Figure and Table

Supplementary Information Supplementary Dataset S1

## Figures and Tables

**Table 1:** Significant differences in enriched pathways for differentially expressed genes

Pathway	No. of differentially expressed genes	P value	Pathway ID
Fatty acid biosynthesis	4	0.0007	hsa00061
Glycine, serine and threonine metabolism	6	0.0012	hsa00260
Metabolic pathways	33	0.0023	hsa01100
Carbon-nitrogen ligase activity, with glutamine as amido-N-donor	1	0.0070	GO:0016884
Hedgehog signalling pathway	1	0.0098	dme04340

**Table 2:** Partially differentially expressed genes involved in significantly enriched pathways

Gene ID	Gene name	Log2 Ratio	Up-down regulation	P value	FDR	Description	Enriched pathway
RIFA001	*FAAH*	11.39	Up	3.63E-05	3.72E-04	Fatty acid amide hydrolase 2	Carbon-nitrogen ligase activity, with glutamine as amido-N-donor
RIFA247	*PKA*	-1.49	Down	1.16E-15	4.47E-14	Protein kinase DC2	Hedgehog signalling pathway
RIFA181	*FAT1*	-2.05	Down	6.38E-08	1.08E-06	Fatty acid synthase	Fatty acid biosynthesis & Metabolic pathways
RIFA215	*FAT2*	-1.71	Down	1.56E-14	5.68E-13	Fatty acid synthase	Fatty acid biosynthesis & Metabolic pathways
RIFA253	*FAT3*	-1.46	Down	2.27E-07	3.54E-06	Fatty acid synthase	Fatty acid biosynthesis & Metabolic pathways
RIFA337	*FAT4*	-1.12	Down	8.57E-06	0.0001	Fatty acid synthase	Fatty acid biosynthesis & Metabolic pathways

**Table 3:** Correlations of expression patterns between genes (N* = 21)

		*Si-CSP9*	*PKA*	*FAAH*	*FAT1*	*FAT2*	*FAT3*	*FAT4*
*Si-CSP9*	Pearson Correlation	1	0.710**	0.663**	0.751**	0.851**	0.690**	0.741**
	Sig. (2-tailed)		0.000	0.001	0.000	0.000	0.001	0.000
*PKA*	Pearson Correlation	0.710**	1	0.980**	0.981**	0.954**	0.995**	0.978**
	Sig. (2-tailed)	0.000		0.000	0.000	0.000	0.000	0.000
*FAAH*	Pearson Correlation	0.663**	0.980**	1	0.987**	0.947**	0.994**	0.991**
	Sig. (2-tailed)	0.001	0.000		0.000	0.000	0.000	0.000
*FAT1*	Pearson Correlation	0.751**	0.981**	0.987**	1	0.983**	0.988**	0.995**
	Sig. (2-tailed)	0.000	0.000	0.000		0.000	0.000	0.000
*FAT2*	Pearson Correlation	0.851**	0.954**	0.947**	0.983**	1	0.956**	0.977**
	Sig. (2-tailed)	0.000	0.000	0.000	0.000		0.000	0.000
*FAT3*	Pearson Correlation	0.690**	0.995**	0.994**	0.988**	0.956**	1	0.989**
	Sig. (2-tailed)	0.001	0.000	0.000	0.000	0.000		0.000
*FAT4*	Pearson Correlation	0.741**	0.978**	0.991**	0.995**	0.977**	0.989**	1
	Sig. (2-tailed)	0.000	0.000	0.000	0.000	0.000	0.000	

N*: number of tested samples; **Correlation is significant at the 0.01 level.

**Table 4:** Primer information

Primer name	Primer sequence (5′-3′)	Product size
*Si-CSP9*-race	F:CAACTGAACATAGCCCTGAGCGACAAR: ACTTTTCAAACGACGTCCGACGGGG	
q*Si-CSP9*	F: GGTCTCCGACGAACAACTR: GAACCAGCGGCACTAAAC	131 bp
q*Si-CSP2*	F: GACGTTGTGCGACAGAAAGCR: TCCAAGTATCGGGTTGGTTCT	187 bp
q*Si-CSP3*	F: GCAATGAGCGTACTGACGTGR: TGCTGTTCTAGTGTGCACGG	152 bp
RNAi-*Si-CSP9*	5′-GATCACTAATACGACTCACTATAGGGCAGGATAGTGCAACAATACTT-3′3′-CTAGTGATTATGCTGAGTGATATCCCGTCCTATCACGTTGTTATGAA-5′5′-AACAGGATAGTGCAACAATACCCCTATAGTGAGTCGTATTAGTGATC-3′3′-TTGTCCTATCACGTTGTTATGGGGATATCACTCAGCATAATCACTAG-5′	
RNAi-*PKA*	5′-GATCACTAATACGACTCACTATAGGGTTTACGAGATGTTGGCGGGTT-3′3′-CTAGTGATTATGCTGAGTGATATCCCAAATGCTCTACAACCGCCCAA-5′5′-AATTTACGAGATGTTGGCGGGCCCTATAGTGAGTCGTATTAGTGATC-3′3′-TTAAATGCTCTACAACCGCCCGGGATATCACTCAGCATAATCACTAG-5′	
q*PKA*	GTAGACTGGTGGGCGTTAGGCG TCTTCGTTCTGTCGGCGATCAA	
q*FAT1*	F:CTGCGACGGTCCCATGTATTR:TGTCATTCGTGCCCACAGTT	177 bp
q*FAT2*	F:GGCTAAGAATTTTTCAGGACGCR:TTTTCCTTGCCAGGTCTACTGT	159 bp
q*FAT3*	F:CAGAAAGCGAAGCATGCGAAR:TGACAAACCGCAACTCTCGT	150 bp
q*FAT4*	F:AATGGGGTGCGATTGGTGATR:CTGCTTACGATAGGCCGGTT	154 bp
q*FAAH*	F:TCCCATACTGCGATGACACGR:TATTCGCCTCAAGTCCACCG	152 bp
