# Supplementary material for: Si-CSP9 regulates the integument and moulting process of larvae in the red imported fire ant, Solenopsis invicta
Source: Sci Rep. 2015 Mar 18;5:9245. doi: 10.1038/srep09245 (PMC4363891; doi:10.1038/srep09245)
Supplement: Supplementary Information — Supplementary Figure and Table [file srep09245-s1.pdf]

Functions of Si-CSP: Si-CSP9 regulates the integument and molting process of larvae in the red imported fire ant, Solenopsis invicta

Daifeng Cheng   Yongyue Lu   Ling Zeng   Xiaofang He*   Guangwen Liang*

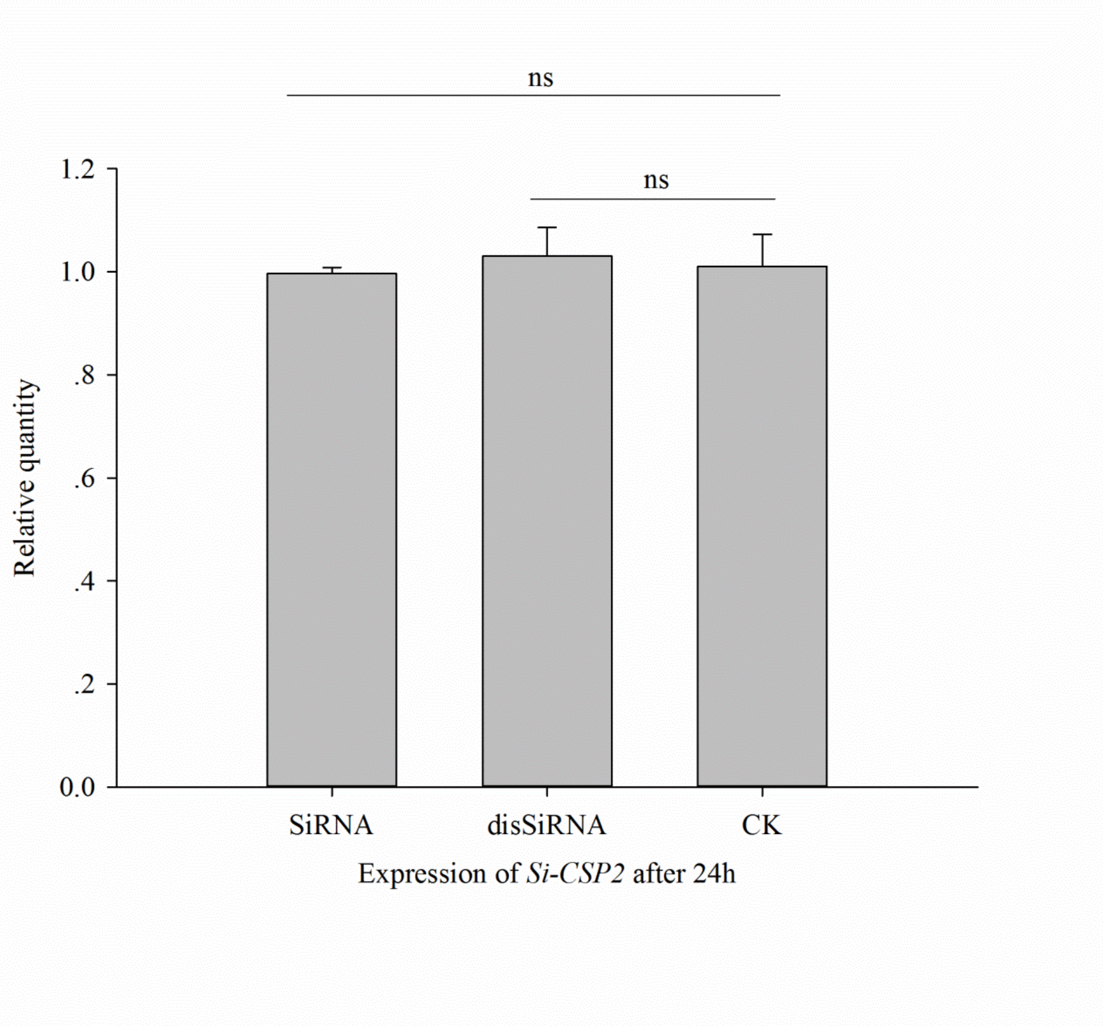

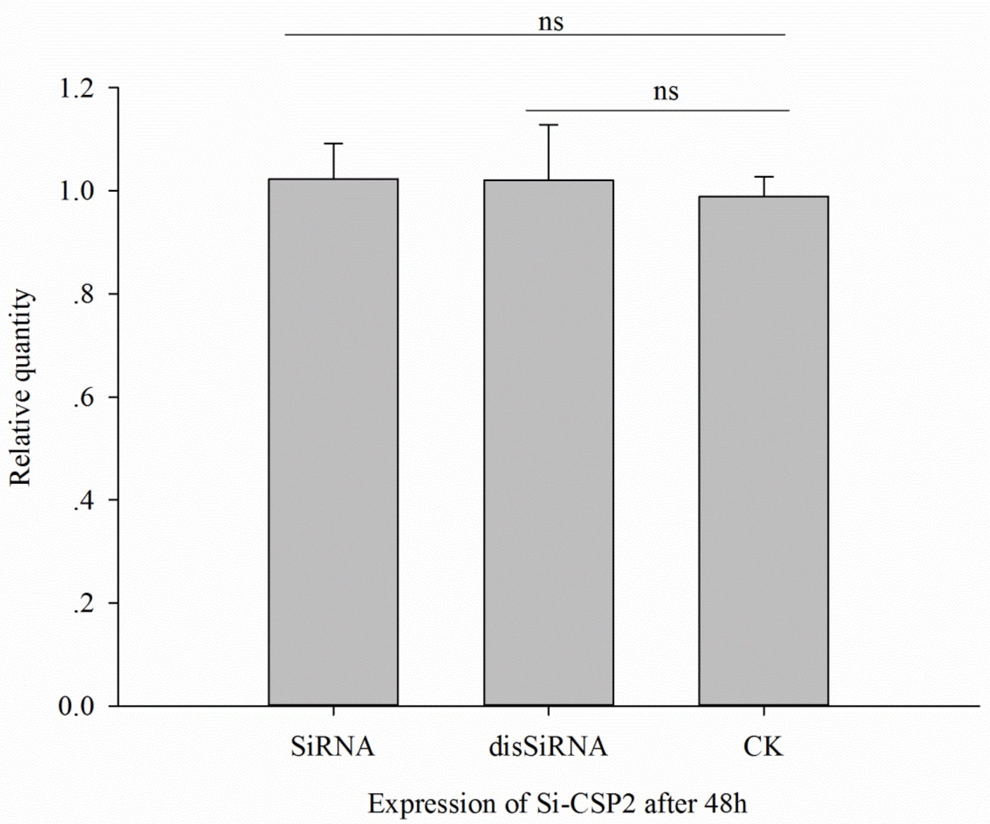

ns
ns
1.2
1.0
.8
.6
.4
.2
0.0
Relative quantity
SiRNA
disSiRNA
CK
Expression of Si-CSP2 after 48h

5

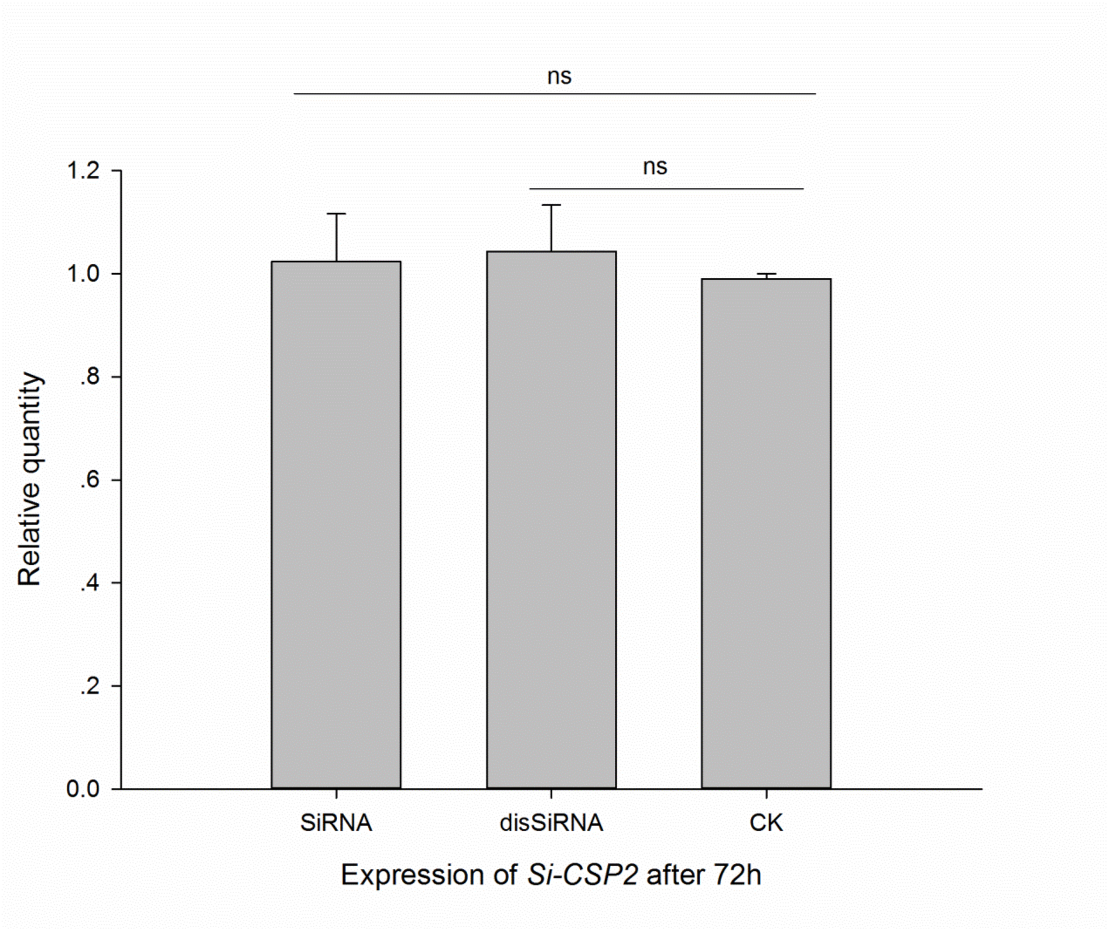
ns
ns
1.2
1.0
.8
.6
.4
.2
0.0
Relative quantity
SiRNA
disSiRNA
CK
Expression of Si-CSP2 after 72h

6

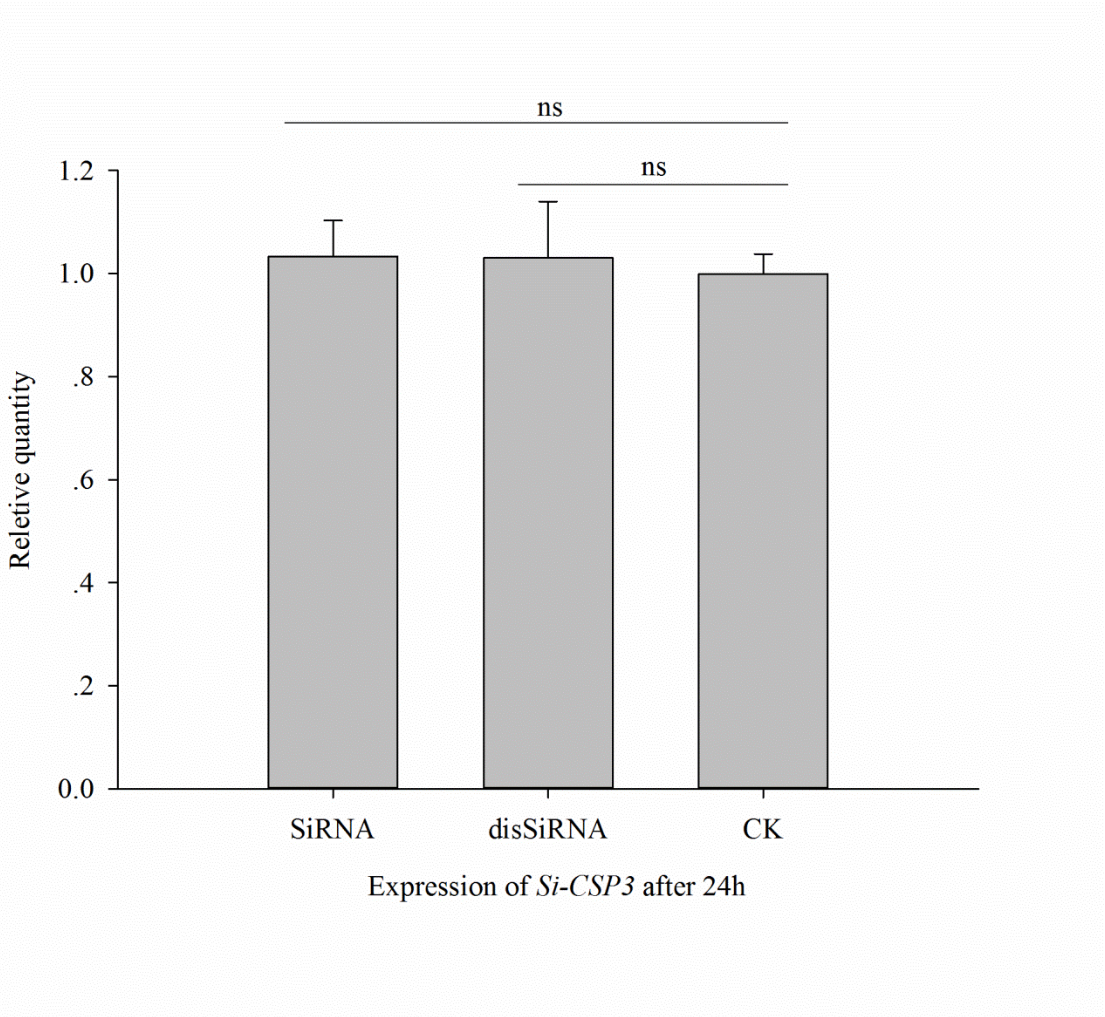
ns
ns
1.2
1.0
.8
.6
.4
.2
0.0
Reletive quantity
SiRNA
disSiRNA
CK
Expression of *Si-CSP3* after 24h

7

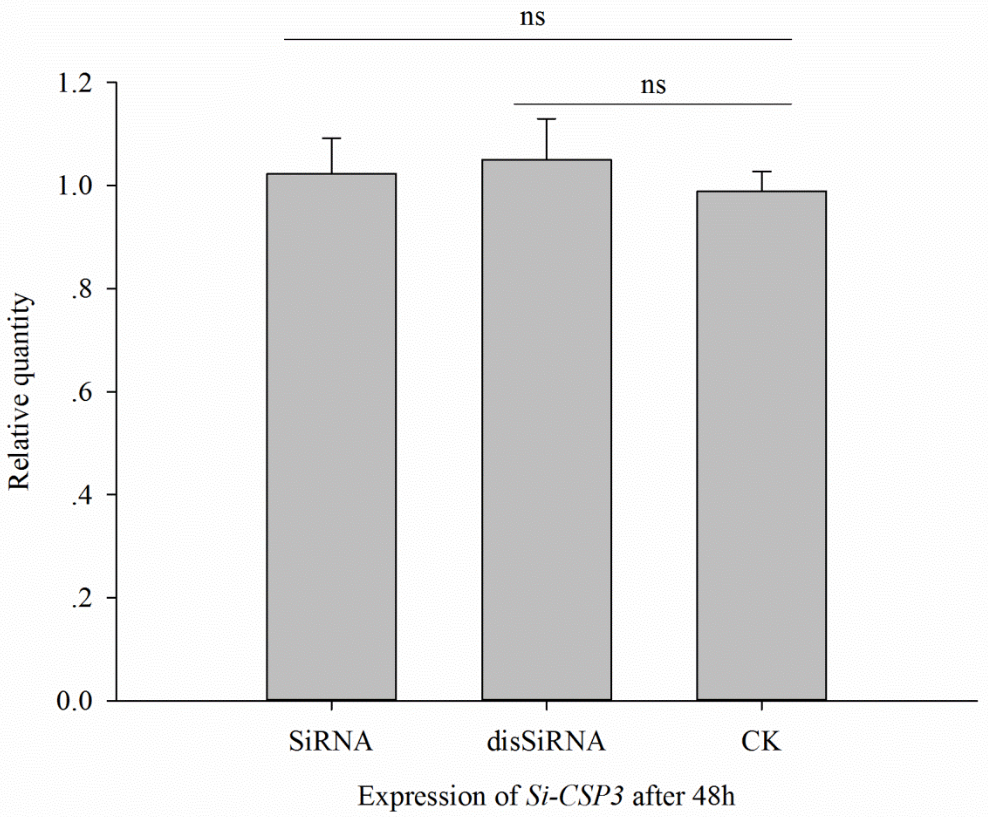
ns
ns
1.2
1.0
.8
.6
.4
.2
0.0
Relative quantity
SiRNA
disSiRNA
CK
Expression of Si-CSP3 after 48h

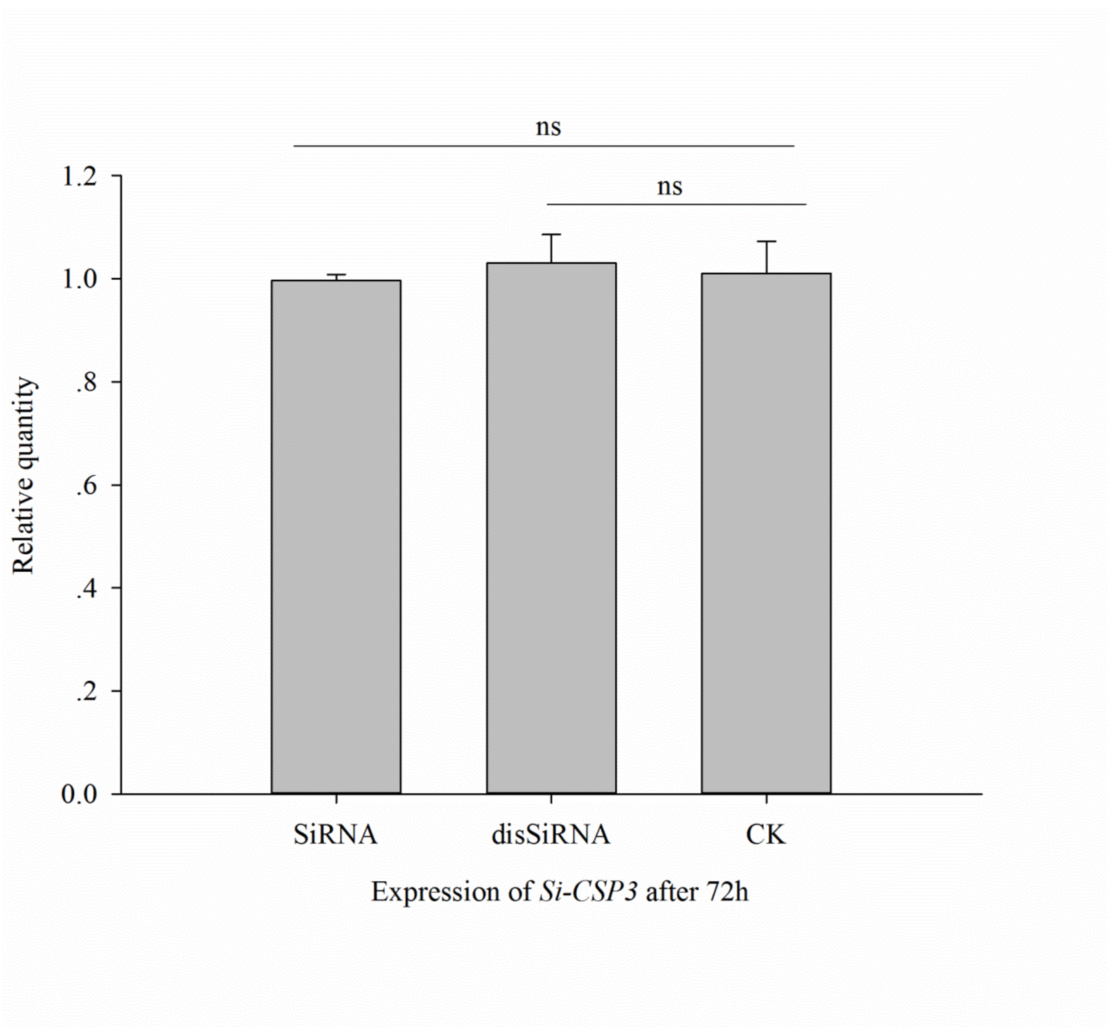

Supplementary Figure S1 Expression of *Si-CSP2* and *Si-CSP3* after *Si-CSP9* being silenced.

SupplementaryTable S1 Genes used in phylogenetic tree reconstruction

	Order	*Species*	*Name*	*Accession number*
PKA	*Diptera*	*Muscadomestica*	*MdomXP_005178559*	*XP_005178559*
	Diptera	*Ceratitiscapitata*	*CcapXP_004535521*	*XP_004535521*
	Diptera	*Drosophila melanogaster*	*DmelAAM50541*	*AAM50541*
	Hymenoptera	*Harpegnathossaltator*	*HsalEFN86858*	*EFN86858*
	Hymenoptera	*Nasoniavitripennis*	*NvitXP_001603535*	*XP_001603535*
	Hymenoptera	*Acromyrmex_echinatior*	*AechEGI70994*	*EGI70994*
	Hymenoptera	*Camponotus_floridanus*	*CfloEFN64599*	*EFN64599*
	Hymenoptera	*Apismellifera*	*AmelXP_393711*	*XP_393711*
	Hymenoptera	*Solenopsisinvicta*	*PKA*	*EFZ22638*
	Lepidoptera	*Bombyxmori*	*BmorXP_004929251*	*XP_004929251*
	Lepidoptera	*Mamestrabrassicae*	*MbraAFP95722*	*AFP95722*

FAT	*Hymenoptera*	*Solenopsisinvicta*	*FAT1*	*EFZ12838*
	Hymenoptera	*Solenopsisinvicta*	*FAT2*	*EFZ11472*
	Hymenoptera	*Solenopsisinvicta*	*FAT3*	*EFZ16301*
	Hymenoptera	*Solenopsisinvicta*	*FAT4*	*EFZ11482*
	Hymenoptera	*Acromyrmexechinatior*	*AechiEGI66297*	*EGI66297*
	Hymenoptera	*Camponotusfloridanus*	*CfloEFN75053*	*EFN75053*
	Hymenoptera	*Harpegnathossaltator*	*HsalEFN81866*	*EFN81866*
	Hymenoptera	*Apismellifera*	*AmelXP_395426*	*XP_395426*
	Hymenoptera	*Apisflorea*	*AfloXP_003690209*	*XP_003690209*
	Hymenoptera	*Bombusterrestris*	*BterNP_001267840*	*NP_001267840*
	Homoptera	*Acyrthosiphonpisum*	*ApisXP_001949072*	*XP_001949072*
	Diptera	*Muscadomestica*	*MdomXP_005189399*	*XP_005189399*
	Diptera	*Ceratitiscapitata*	*CcapXP_004524714*	*XP_004524714*
	Diptera	*Aedesaegypti*	*AaegXP_001654967*	*XP_001654967*
	Coleoptera	*Triboliumcastaneum*	*TcasXP_970384*	*XP_970384*
FAAH	*Hymenoptera*	*Solenopsisinvicta*	*FAAH*	*EFZ09899*
	Hymenoptera	*Acromyrmexechinatior*	*AechEGI60868*	*EGI60868*
	Hymenoptera	*Harpegnathossaltator*	*HsalEFN75981*	*EFN75981*
	Hymenoptera	*Apisflorea*	*AfloXP_003698394*	*XP_003698394*
	Lepidoptera	*Bombyxmori*	*BmorXP_004923963*	*XP_004923963*
	Lepidoptera	*Bombyxmori*	*BmorXP_004924016*	*XP_004924016*
	Lepidoptera	*Bombyxmori*	*BmorXP_004931785*	*XP_004931785*
	Diptera	*Muscadomestica*	*MdomXP_005185712*	*XP_005185712*
	Diptera	*Ceratitiscapitata*	*CcapXP_004536939*	*XP_004536939*
	Diptera	*Ceratitiscapitata*	*CcapXP_004523064*	*XP_004523064*
	Homoptera	*Acyrthosiphonpisum*	*ApisXP_001946922*	*XP_001946922*
	Coleoptera	*Anoplophoraglabripennis*	*AglaJAB65838*	*JAB65838*
